# Enzymatic Cleavage of Type II Restriction Endonucleases on the 2′-O-Methyl Nucleotide and Phosphorothioate Substituted DNA

**DOI:** 10.1371/journal.pone.0079415

**Published:** 2013-11-15

**Authors:** Guojie Zhao, Jun Li, Zhaoxue Tong, Bin Zhao, Runqing Mu, Yifu Guan

**Affiliations:** 1 Key Laboratory of Medical Cell Biology, Ministry of Education, Department of Biochemistry and Molecular Biology, China Medical University, Shenyang, Liaoning, China; 2 Department of Movement Science, Shenyang Sport University, Shenyang, Liaoning, China; 3 Department of Laboratory Medicine, First Affiliated Hospital, China Medical University, Shenyang, Liaoning, China; University of Pittsburgh, United States of America

## Abstract

The effects of nucleotide analogue substitution on the cleavage efficiencies of type II restriction endonucleases have been investigated. Six restriction endonucleases (EcoRV, SpeI, XbaI, XhoI, PstI and SphI) were investigated respectively regarding their cleavage when substrates were substituted by 2′-O-methyl nucleotide (2′-OMeN) and phosphorothioate (PS). Substitutions were made in the recognition sequence and the two nucleotides flanking the recognition sequence for each endonuclease. The endonuclease cleavage efficiencies were determined using FRET-based assay. Results demonstrated a position-dependent inhibitory effect of substitution on the cleavage efficiency for all the six endonucleases. In general, the 2′-OMeN substitutions had greater impact than the PS substitutions on the enzymatic activities. Nucleotides of optimal substitutions for protection against RE cleavage were identified. Experimental results and conclusions in this study facilitate our insight into the DNA-protein interactions and the enzymatic cleavage mechanism, particularly for those whose detailed structure information is not available. In addition, the information could benefit the development of bioengineering and synthetic biology.

## Introduction

Type II restriction endonucleases (REs) represent a special group of enzymes. Working along with methylases, they constitute a perfect native defense system to prevent the prokaryotic host genomes from attacks of foreign nucleic acids [1], [2], [3]. This protection is accomplished by hydrolysis of the phosphoester linkage of the invading DNA strands by type II REs since they are highly specific to recognize short palindromic sequences of 4–8 bp and to cleave one phosphoester bond at a specific site within the recognition sequences. This hydrolysis leaves behind two pieces of dsDNA fragments containing either 3′-overhangs, or 5′-overhangs or blunt ends depending upon the nature of the endonucleases. Thousands of type II REs have been discovered to date. However, only a few have been thoroughly characterized regarding their structures, enzymatic kinetics and cleavage mechanisms. The majority of type II REs remains unexplored due primarily to the absence of structural information. To identify particular molecular interactions between REs and their duplex substrates and to obtain an understanding about the nuclease cleavage mechanisms, selective substitutions of nucleotide analogues for particular nucleotides of RE recognition sequences have been utilized extensively [4], [5], [6], [7].

Three types of nucleotide analogues have been widely used for the purpose of probing molecular interactions in the nuclease cleavage. Base-modified nucleotides in DNA duplexes can alter the base pair geometry, thus, extruding the base pairs into either the minor groove or the major groove and disturbing the hydrogen bond formation with nearby amino acid residues of proteins [8], [9]. Modifications on the phosphate group provide more flexible backbone structures which can comfort the RE structures [10], [11]. Furthermore, sugar modifications provide more advantages in distinguishing the molecular interaction patterns. The modifications at the C2′ atom constrain the ribosyl ring structure to two particular conformations: the C2′-endo sugar pucker conformation which is the energetically favorable structure in B-form DNA, and the C3′-endo sugar pucker conformation which is frequently observed in A-form DNA and RNA. Thus, the C2′-modified nucleotides can be used as the DNA mimics and RNA mimics as well. In addition, when incorporated into the oligonucleotides, they still obey the canonical rules of complementary base pair formation. The substituents at the C2′ atom have a wide range of choices to offer differences in electronegativity, hydrophobicity, hydrogen bond forming capability and steric hindrance [6], [12].

In our previous studies, nucleotide derivatives have shown that the substitutions could reveal the network of the enzyme-substrate interactions. Beyond that, these nucleotide analogues have the potential to protect the recognition sequence from nuclease cleavage, and can be used in many nucleic acid related techniques, such as protection of the circular DNA template in RCA (rolling circle amplification) [13] and making sticky ends in genetic cloning [14], [15]. Most importantly, protection of REs has been applied to therapeutic treatment of certain diseases caused by DNA mutations [16]. Currently, these restriction endonucleases have evolved into powerful tools in genetic engineering and synthetic biology. Exploring the interaction of these nucleotide analogues with endonucleases is of extreme importance in fundamental researches as well as in biomedical applications.

In this study, we investigated the effects of the nucleotide analogues substitutions on the endonuclease cleavage of six REs: EcoRV, PstI, SpeI, SphI, XbaI and XhoI. These six REs were chosen based on the considerations that they are representatives of a particular group of REs. EcoRV will create a blunt end, XhoI, XbaI and SpeI give rise to a 5′-overhang, whereas SphI and PstI generate a 3′-overhang. Among them, XbaI and SpeI are special since they are a pair of isocaudomers. Although their recognition sequences are different, they will create the identical 5′-overhangs after cleavage. Thereafter, these two 5′-overhangs can be rejoined to form a new sequence referred to as a linkage scar which cannot be digested by either of these two enzymes. This approach is truly significant in synthetic biology.

Two types of nucleotide analogues were used in the current study. The first one was 2′-O-methyl nucleotide (2′-OMeN), where the –OH group on the C2′ atom is replaced by a –OCH_3_ group. The 2′-OMeN has a constrained C3′-endo pucker conformation and exhibit many unique features, such as an enhanced binding affinity toward their complementary RNA targets [17], [18], [19], an improved identification of mismatched base pairs [20], a resistance against the exonuclease digestion [21], [22], fast hybridization kinetics [23], and the capability of modulating structure transitions of the G-quadruplexes [24]. The second one was phosphorothioate where one phosphoryl oxygen atom of the DNA backbone is substituted by a sulfur atom. Such substitution affects the electron density distribution as well as the hydrogen bond formation [25]. Our results showed that nucleotide derivatives substitutions demonstrated a strong position-dependent behavior of the endonuclease cleavage, presenting good candidates for RE’s cleavage protection as well as cleavage enhancement used in genetic engineering and synthetic biology.

## Materials and Methods

### Preparation of Oligonucleotides and Restriction Endonucleases

**Figure 1:**
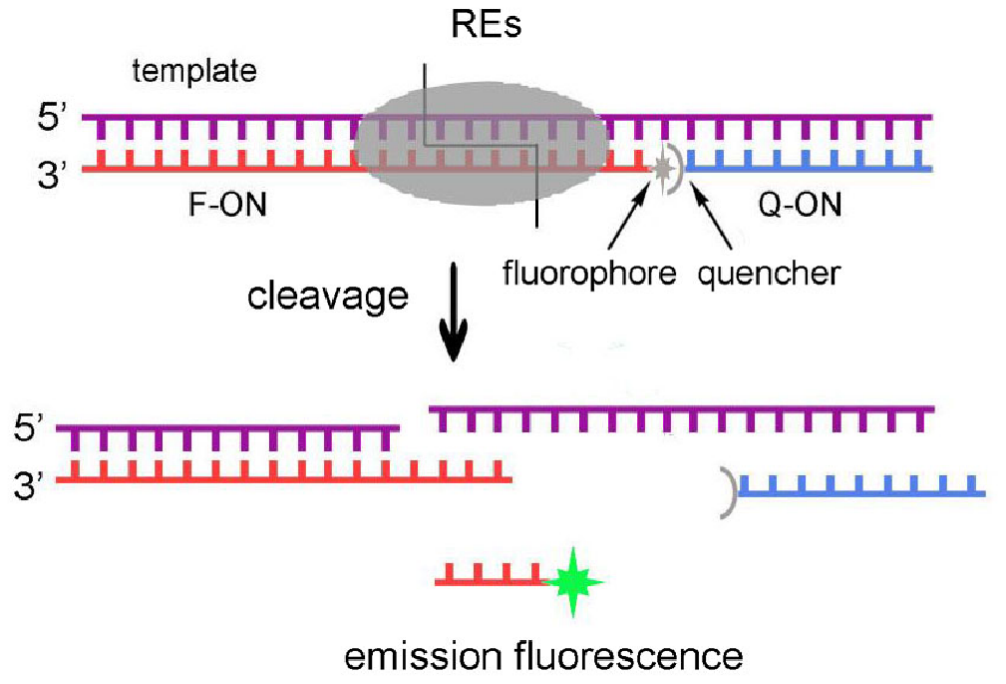
Schematic diagram of FRET assay for evaluating the cleavage efficiency of restriction endonucleases.

In this experiment, three types of oligonucleotides (template, F-ON, Q-ON respectively) have been used to form duplex substrates. The template was 45-nt long containing the RE recognition sequence. The F-ON was 26-nt long with a fluorophore (FITC) at the 5′-end. The Q-ON was 17-nt long with a quencher (Dabcyl) at the 3′-end. By complementing to the template, the F-ON and the Q-ON were arranged so closely that fluorescence signals could be quenched before cleavage (Figure 1). The sequences of oligonucleotides used were listed in Table 1.

**Table 1:** Oligonucleotides used in this study.

Name	Sequence
EcoRV-template	5′-ATACGCATACCTG T**GATATC**T GGCTAAAAGCACACGCACGGAGAC-3′
EcoRV-T(−1)	5′-ATACGCATACCTG T **GATATC**T GGCTAAAAGCACACGCACGGAGAC-3′
EcoRV-G1	5′-ATACGCATACCTG T**GATATC**T GGCTAAAAGCACACGCACGGAGAC-3′
EcoRV-A2	5′-ATACGCATACCTG T**GATATC**T GGCTAAAAGCACACGCACGGAGAC-3′
EcoRV-T3	5′-ATACGCATACCTG T**GATATC**T GGCTAAAAGCACACGCACGGAGAC-3′
EcoRV-A4	5′-ATACGCATACCTG T**GATATC**T GGCTAAAAGCACACGCACGGAGAC-3′
EcoRV-T5	5′-ATACGCATACCTG T**GATATC**T GGCTAAAAGCACACGCACGGAGAC-3′
EcoRV-C6	5′-ATACGCATACCTG T**GATATC**T GGCTAAAAGCACACGCACGGAGAC-3′
EcoRV-T7	5′-ATACGCATACCTG T**GATATC** T GGCTAAAAGCACACGCACGGAGAC-3′
EcoRV-PS1	5′-ATACGCATACCTG T**pGATATC**T GGCTAAAAGCACACGCACGGAGAC-3′
EcoRV-PS2	5′-ATACGCATACCTG T**GpATATC**T GGCTAAAAGCACACGCACGGAGAC-3′
EcoRV-PS3	5′-ATACGCATACCTG T**GApTATC**T GGCTAAAAGCACACGCACGGAGAC-3′
EcoRV-PS4	5′-ATACGCATACCTG T**GATpATC**T GGCTAAAAGCACACGCACGGAGAC-3′
EcoRV-PS5	5′-ATACGCATACCTG T**GATApTC**T GGCTAAAAGCACACGCACGGAGAC-3′
EcoRV-PS6	5′-ATACGCATACCTG T**GATATpC**T GGCTAAAAGCACACGCACGGAGAC-3′
EcoRV-PS7	5′-ATACGCATACCTG T**GATATCp**T GGCTAAAAGCACACGCACGGAGAC-3′
SpeI-template	5′-ATACGCATACCTG T**ACTAGT**T GGCTAAAAGCACACGCACGGAGAC-3′
XbaI-template	5′-ATACGCATACCTG T**TCTAGA**T GGCTAAAAGCACACGCACGGAGAC-3′
XhoI-template	5′-ATACGCATACCTG T**CTCGAG**T GGCTAAAAGCACACGCACGGAGAC-3′
PstI-template	5′-ATACGCATACCTG T**CTGCAG**T GGCTAAAAGCACACGCACGGAGAC-3′
SphI-template	5′-ATACGCATACCTG T**GCATGC**T GGCTAAAAGCACACGCACGGAGAC-3′
EcoRV-F-ON	FITC-5′-TAGCC A**GATATC**A CAGGTATGCGTAT-3′
SpeI-F-ON	FITC-5′-TAGCC A**ACTAGT**A CAGGTATGCGTAT-3′
XbaI-F-ON	FITC-5′-TAGCC A**TCTAGA**A CAGGTATGCGTAT-3′
XhoI-F-ON	FITC-5′-TAGCC A**CTCGAG**A CAGGTATGCGTAT-3′
PstI-F-ON	FITC-5′-TAGCC A**CTGCAG**A CAGGTATGCGTAT-3′
SphI-F-ON	FITC-5′-TAGCC A**GCATGC**A CAGGTATGCGTAT-3′
Q-ON	5′-GTCTCCGTGCGTGTGCT-3′-DABCYL

The bold faced are the endonuclease recognition sequences. The underlined are the 2′-OMeN modified nucleotides, and letter p represents the phosphothioate modification. For simplicity, only the template sequences of endonucleases PstI, XhoI, XbaI, SpeI and SphI are listed, and the modified sequences were not included.

Nucleotide analogue 2′-OMeN or PS was used to substitute the recognition sequences of restriction endonucleases. These oligonucleotides at HPLC grade were purchased from Sangon Biotech Co. Ltd. (Shanghai, China), and were prepared at the concentration of 100 µM in ddH_2_O without further purification. These stock solutions were stored in −20°C. Six restriction endonucleases were purchased from TaKaRa Biotechnology Company (Dalian, China). Their stock concentrations were 15 U/µl for EcoRV (Cat # : D1042A), 15 U/µl for PstI (Cat # : D1073A), 10 U/µl for XhoI (Cat # : D1094A), 15 U/µl for XbaI (Cat # : D1093A), 10 U/µl for SpeI (Cat # : D1086A), and 10 U/µl for SphI (Cat # : D1180A), respectively. EcoRV creates a blunt end, XbaI, SpeI and XhoI generate a 5′-overhang, and SphI and PstI create a 3′-overhang.

### FRET Assay for Enzymatic Cleavage

The duplex substrate of enzyme is composed of the template, F-ON and Q-ON at the same concentration of 50 nM in 100 µl buffer solution containing 50 mM Tris-Cl, 10 mM MgCl_2_, 1 mM DTT, and 100 mM NaCl (pH 7.5). The template and F-ON were mixed first, and fluorescence was measured as F0. After adding Q-ON, stable duplex substrates were formed. Since the quencher and the fluorophore were so close in space that the fluorescence signals were quenched due to the fluorescence energy transfer, and the low fluorescence signal was defined as F1. Once restriction endonuclease was added into the substrate solution, it bound to the recognition sequence and started to cleave the sequence formed by F-ON and the template. The cleaved 5′-portion of F-ON was too short to hybridize with the template and then was released, generating a fluorescence signal Ft (Figure 1). The relative fluorescence intensity (RF) was defined as RF = (Ft–F1)/(F0–F1).

In this experiment, fluorescence signals were recorded at 37°C on Microplate Reader (Infinite M200, Tecan, USA) with excitation wavelength at 480 nm and emission wavelength at 524 nm. The signals were collected every 15 seconds with an integration time of 20 microseconds, and measurements were performed by monitoring the fluorescence emission with time over 20 min.

## Results and Discussion

Restriction endonucleases can be used as a group of representative models for investigating the DNA-protein interactions dictating biological events since the conserved recognition sequences could build an interaction network with particular amino acid residues at the catalytic center of these restriction endonucleases. However, lack of the detailed structural information of X-ray crystallography and NMR spectroscopy has made it very difficult to view these interactions directly. Thus, nucleotide analogues have been utilized to substitute DNA nucleotides at the selected positions, and demonstrated different cleavage behavior due to particular interactions. Nucleotide substitution has become excellent tools in analyzing the mechanisms of restriction endonucleases binding and cleaving.

### Validation of the FRET Assay

To study the effect of nucleotide analogues on the endonuclease cleavage, we used the FRET-based assay in the present study which has been well established previously for monitoring the restriction endonuclease cleavage activity. This FRET-based analytical platforms can monitor the fluorescence signal changes as the cleavage reaction is progressing, and become a popular approach already due to their advantages of real-time, convenience, cost-effectiveness and high sensitivity [26], [27], [28], [29]. The platform we used here is derived from the FRET assay reported previously [29].

**Figure 2:**
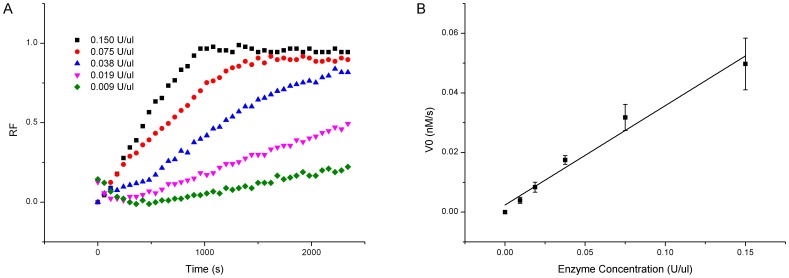
Effects of enzyme concentrations on EcoRV cleavage. (A) Time course plot of fluorescence intensity. (B) Initial velocities affected by enzyme concentrations.

To determine the enzyme concentration adequate for experiments in the current study, we tested the effects of the enzyme concentration on the cleavage efficiency for the un-substituted substrates. The EcoRV concentrations in the range of 0.009 U/µl to 0.15 U/µl were examined. At lower concentrations, the fluorescence intensities increased monotonically as the reaction was progressing, and at higher concentrations, the fluorescence intensities increased rapidly and then became saturated, exhibiting the typical enzymatic behavior (Figure 2A). The initial reaction velocities were then calculated, and showed a linear relationship with the EcoRV concentrations (Figure 2B). Other five endonucleases have also shown similar linearity between the reaction velocities and the endonuclease concentrations (Figure S1–S5). The R^2^ values of six endonucleases were measured to be 0.9742±0.0056. This linearity validates the FRET-based platform for determination of appropriate enzyme concentrations in the following substitution experiments.

### Effect of 2′-O-methyl Nucleotide Substitution

**Figure 3:**
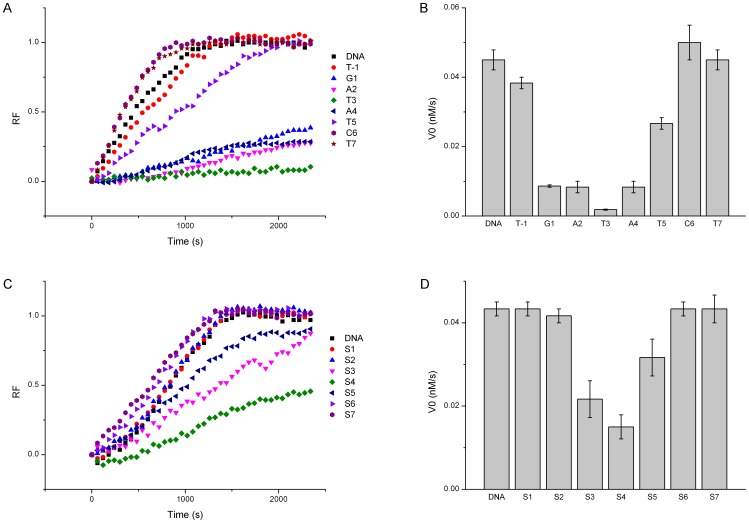
Effects of 2′-OMeN and PS substitution positions on EcoRV cleavage. (A) Time course plot of fluorescence intensity of 2′-OMeN substitution. (B) Initial velocities affected by position-dependent 2′-OMeN substitution. (C) Time course plot of fluorescence intensity of PS substitution. (D) Initial velocities affected by position-dependent PS substitution.

The effect of 2′-OMeN substitution on the endonuclease cleavage demonstrated a strong position-dependent behavior. As shown in Figure 3A, the fluorescence signal changed remarkably when different positions of the EcoRV recognition sequence were substituted by 2′-OMeN, and Figure 3B shows the calculated corresponding initial velocities.

In comparison with un-substituted DNA sequence, 2′-OMeN-substitution at the T3 position of the EcoRV sequence GAT/ATC (referred to as EcoRV-T3) inhibited the cleavage activity almost completely (Figure 3) (The slash in each sequence represents the cleavage site.). 2′-OMeN-substitutions at G1, A2 and A4 positions reduced the enzymatic cleavage significantly, and substitution at T5 position decreased the enzymatic activity moderately. In contrast, the substitution at C6 position enhanced cleavage slightly. The flanking positions (T-1) and T7 of EcoRV were also substituted and their cleavage velocities were close to that of un-substituted substrate with a small margin.

**Figure 4:**
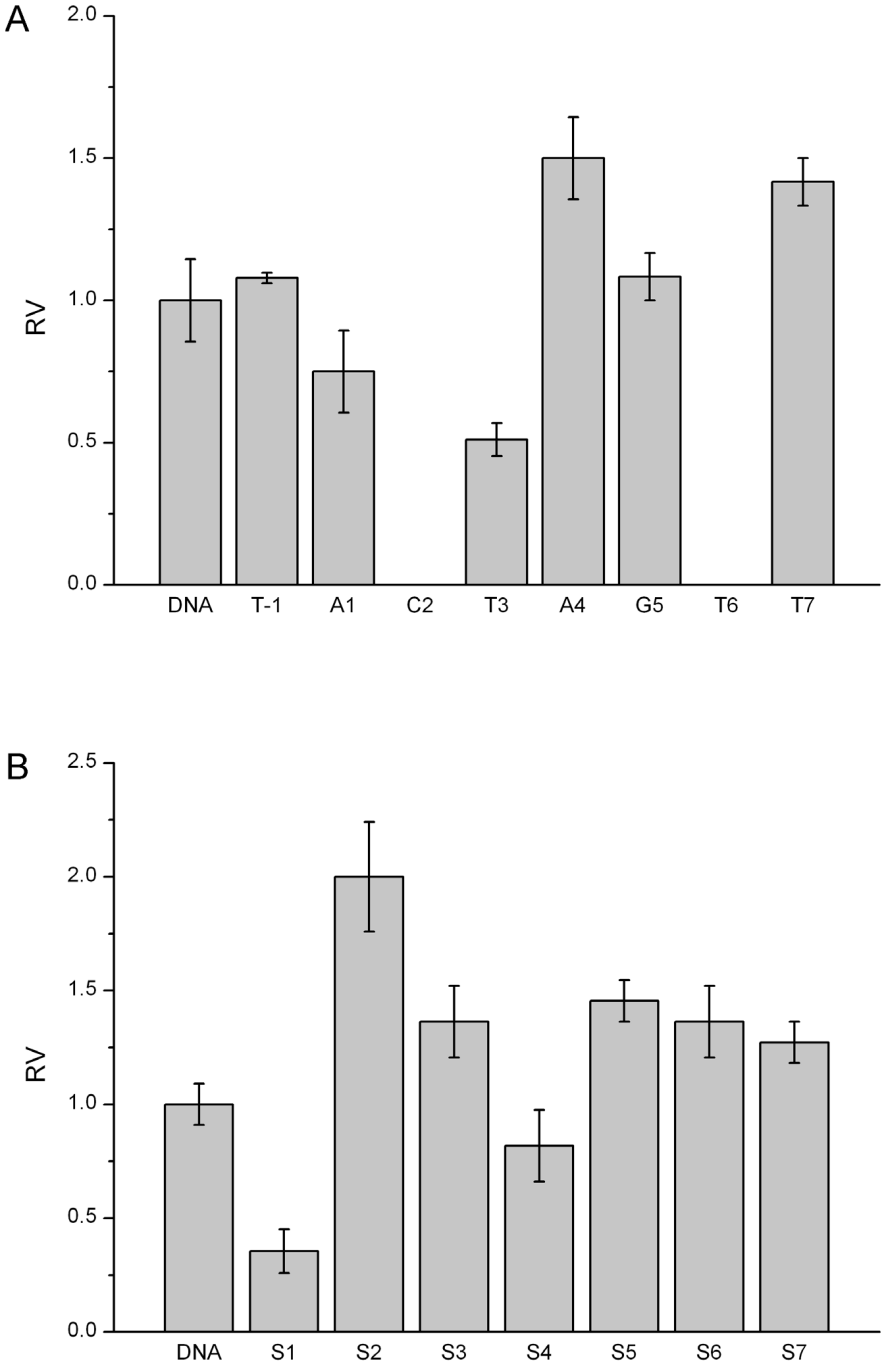
Relative initial velocities (RV) of SpeI cleavage affected by position-dependent substitution. (A) Effects of 2′-OMeN substitution positions. (B) Effects of PS substitution positions.

**Figure 5:**
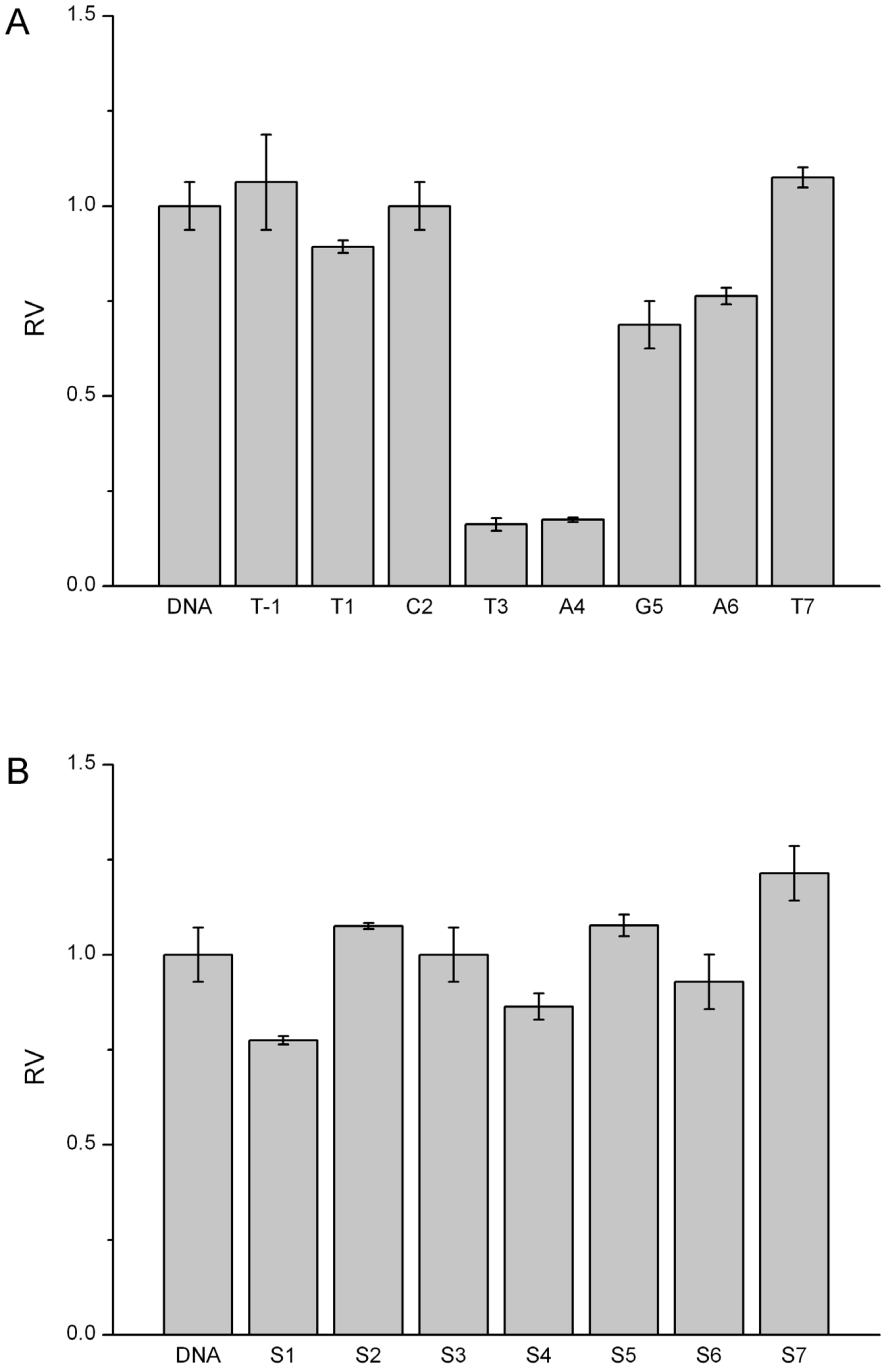
Relative initial velocities (RV) of XbaI cleavage affected by position-dependent substitution. (A) Effects of 2′-OMeN substitution positions. (B) Effects of PS substitution positions.

**Figure 6:**
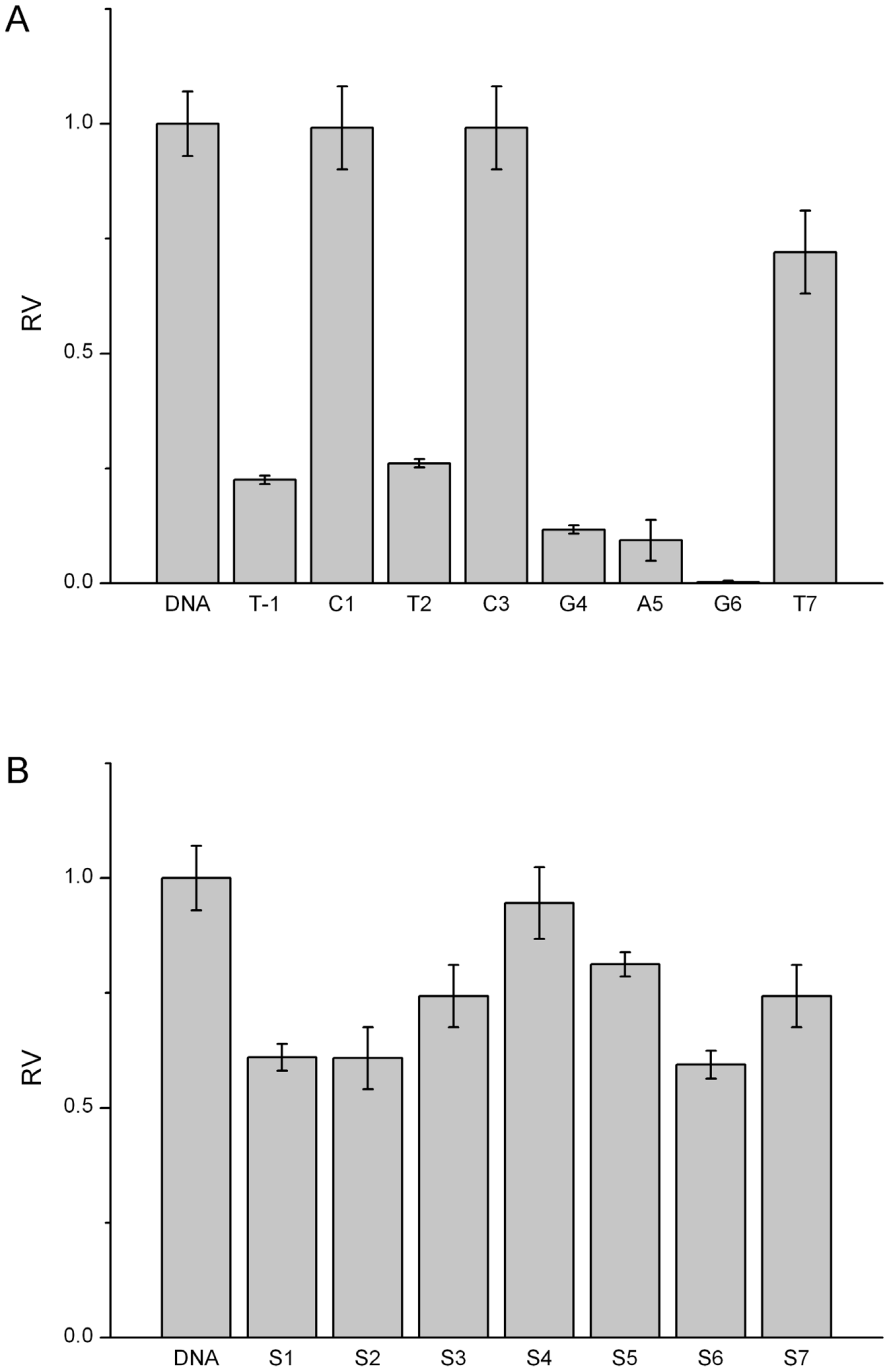
Relative initial velocities (RV) of XhoI cleavage affected by position-dependent substitution. (A) Effects of 2′-OMeN substitution positions. (B) Effects of PS substitution positions.

**Figure 7:**
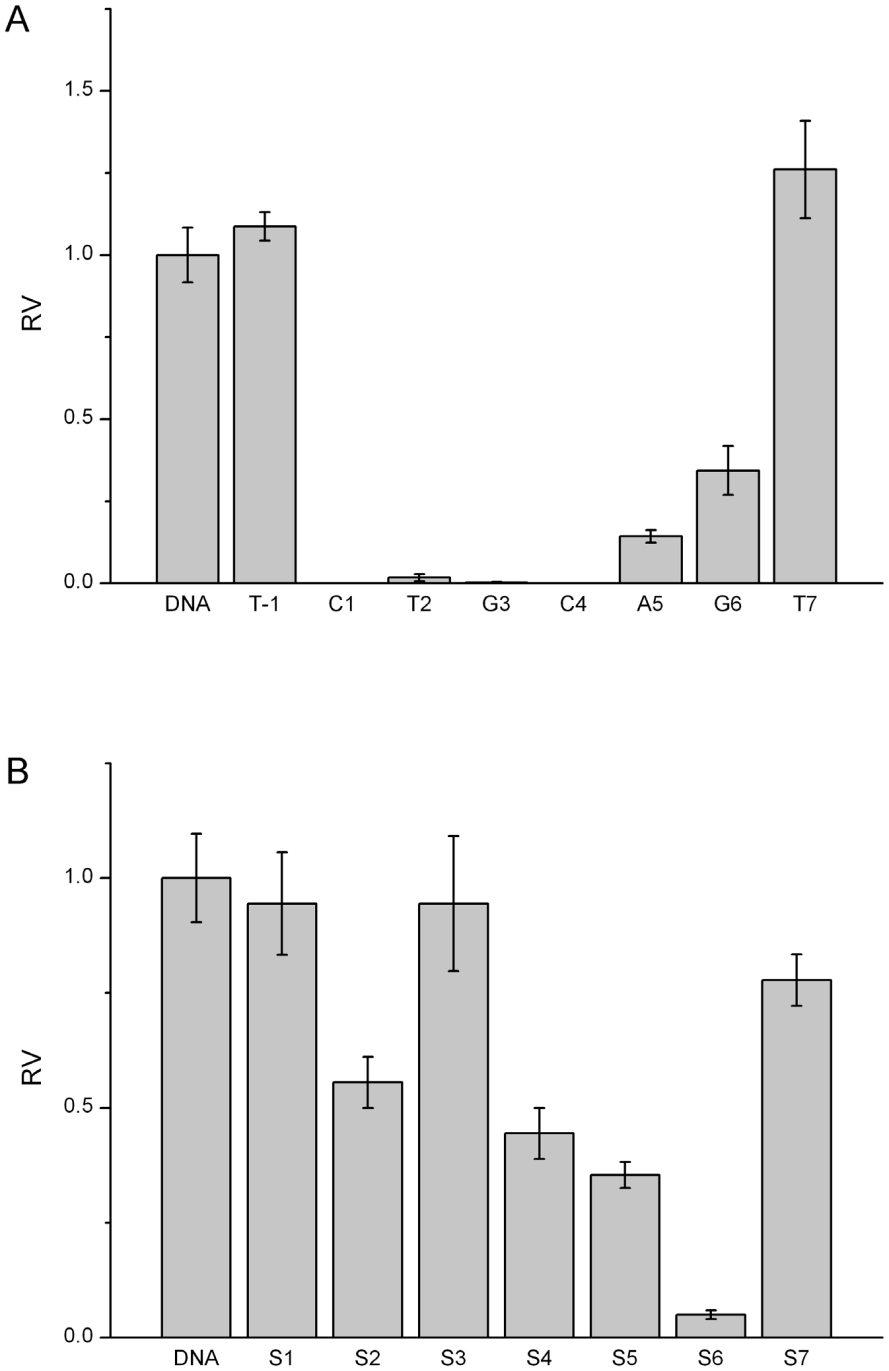
Relative initial velocities (RV) of PstI cleavage affected by position-dependent substitution. (A) Effects of 2′-OMeN substitution positions. (B) Effects of PS substitution positions.

**Figure 8:**
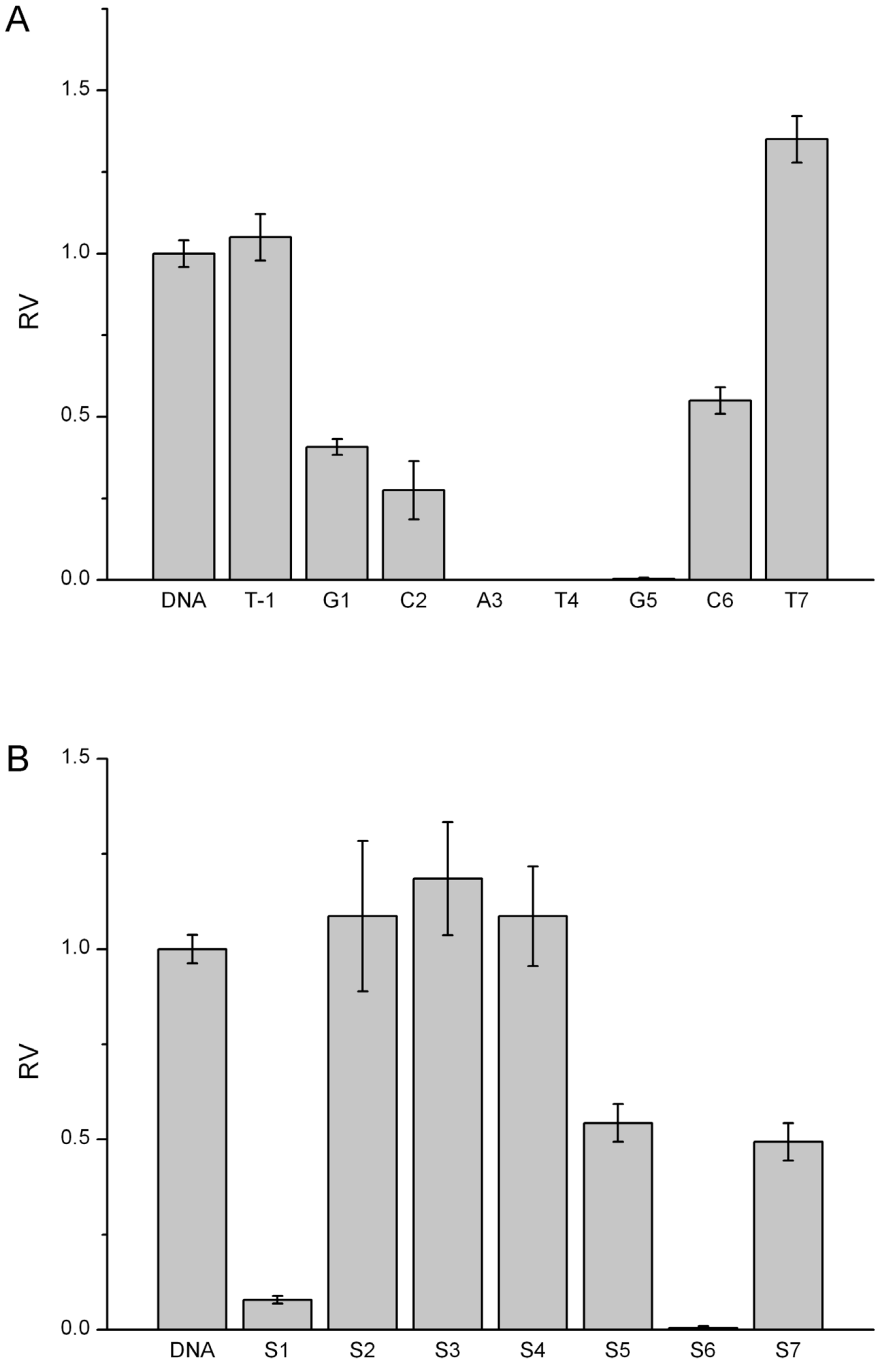
Relative initial velocities (RV) of SphI cleavage affected by position-dependent substitution. (A) Effects of 2′-OMeN substitution positions. (B) Effects of PS substitution positions.

Similarly, other five endonucleases have demonstrated the position-dependent cleavage by 2′-OMeN substitutions. For simplicity, their initial velocities of 2′-OMeN substitutions at eight positions are shown in Figure 4A–8A. We evaluated the substitution effects by comparing the relative ratios of their initial velocities with respect to that of the un-substituted species (Table 2), where the initial cleavage velocities were classified into five categories: completely inhibited cleavage (I), significantly reduced cleavage (S), moderately reduced cleavage (M), no change (N) and enhanced cleavage (E). For instance, substitutions at the T3 position of EcoRV sequence GAT/ATC (Figure 3B), C2 and T6 positions of SpeI sequence A/CTAGT (Figure 4A), A5 and G6 positions of XhoI sequence C/TCGAG (Figure 6A), C1, T2, G3 and C4 positions of PstI sequence C/TGCAG (Figure 7A), A3, T4 and G5 positions of SphI sequence GCATG/C (Figure 8A) led to a complete inhibition of the enzymatic cleavage. Surprisingly, EcoRV-C6 (Figure 3B), SpeI-A4, T7 (Figure 4A), XbaI-T7 (Figure 5A), PstI-T7 (Figure 7A) and SphI-T7 (Figure 8A) showed the enhanced cleavage for the 2′-OMeN-substituted substrates.

**Table 2:** Relative initial cleavage rate of 2′-OMeN modification.

RV	DNA	OMe(−1)	OMe1	OMe2	OMe3	OMe4	OMe5	OMe6	OMe7
EcoRV GAT/ATC	N	M	S	S	I	S	M	E	N
SpeI A/CTAGT	N	N	M	I	M	E	N	I	E
XbaI T/CTAGA	N	N	N	N	S	S	M	M	E
XhoI C/TCGAG	N	S	N	S	N	S	I	I	M
PstI CTGCA/G	N	N	I	I	I	I	S	S	E
SphI GCATG/C	N	N	S	S	I	I	I	M	E

RV (relative velocity) = V0(modified)/V0(unmodified).

RV<0.1: completely inhibited cleavage (I); 0.1<RV<0.5: significantly reduced cleavage (S); 0.5<RV<0.9: moderately reduced cleavage (M); 0.9<RV<1.1: no change (N); 1.1<RV: enhanced cleavage (E).

It is interesting to notice that EcoRV-T3, SpeI-C2 and SphI-G5 are at the cleavage sites. Thus, substitution-induced inhibitory effect could be attributed to the fact that substitution might disturb the interactions with key amino acid residues in the catalytic centers of these endonucleases and repress the cleavage. On the other hand, EcoRV-G1, EcoRV-A2, SpeI-T6, XhoI-A4, XhoI-A5, XhoI-G6, PstI-C1, PstI-T2, PstI-G3, PstI-C4, SphI-A3 and SphI-T4 are not at the cleavage sites, suggesting that these reduced cleavage efficiencies might result from the interference of the endonuclease-substrate binding. Our previous study has shown that 2′-OMeN substituted nucleotides showed a reduced binding affinity toward their complementary DNA nucleotides [30]. The data also suggested that it is not necessary to choose the cleavage site as the substituted position to achieve an effective protection against restriction endonuclease cleavage. Two nucleotide positions (−1) and 7 flanking the cognate sequence have also been examined. Some substitutions increased the cleavage slightly, such as PstI-T7, SpeI-T7, XbaI-T7 and SphI-T7, whereas others reduced the cleavage significantly, such as XhoI-T(−1). It has been known that although these two positions do not participate in the sequence recognition, they do influence the catalytic property [5]. In addition, it was observed that the pair of isocaudomers, SpeI and XbaI, did not show a similar cleavage pattern in spite of they can create the identical 5′-overhang.

These six endonucleases in this study exhibited a large degree of diversity in amino acid compositions and biological functions, and only EcoRV has been well studied both structurally and functionally. To understand their cleavage mechanism, substitutions using base analogues have been examined previously (Table S1). However, use of these base analogues has a limitation since their substitutions need to satisfy the requirement of base pair formation. Thus, sugar and phosphate analogues can make substitutions in a position-dependent manner, providing the feasibility to probe some unknown molecular interactions.

The recognition of the substrate sequence by a restriction endonuclease depends on their interactions between each nucleotide of the recognition sequence and the interacting amino acid residues of endonucleases. The 2′-OMeN substitution for a specified position of the cognate sequence would bring about additional interactions to the RE cleavage. First, the larger steric hindrance of the methoxyl group at the C2′ atom constrains the sugar pucker in a C3′-endo conformation, which alters six torsion angles along the phosphate backbone -P-O5′-C5′-C4′-C3′-O3′-P- of DNA strand. Secondly, this methoxyl group introduces a large hydrophobicity and could alter the interaction network significantly. These two effects might combine together to introduce a potential interference in the catalytic center, resulting in the unexpected cleavage retardation. More thorough analysis requires the detailed structural information.

### Effect of Phosphorothioate Substitution

Replacement of one non-bridging oxygen atom of the -C-O-P(O_2_)-O-C- group by sulfur atom generates an asymmetric -C-O-P(O)(S)-O-C- group. The sulfur atom is slightly larger than the oxygen atom and the P-S bond is slightly longer than the P-O bond. In addition, the negative charge is evenly distributed over the two non-bridging oxygen atoms of the phosphate, whereas the negative charge favors to locate on the sulfur atom in the case of phosphorothioate. Thus, although this replacement does not change the phosphodiester backbone, it indeed alters the electron density distribution, and consequently affects the cleavage behavior.

As shown in Figure 3D, PS substitution for the -T3pA4- phosphate group (referred to as EcoRV-S4) showed the significantly reduced cleavage, and PS substitution for the -A2pT3- and -A4pT5- phosphate group (referred to as EcoRV-S3, S5) showed the moderately reduced cleavage. However, substitutions at other positions showed no effect at all on the cleavage.

Similarly, other five endonucleases have also demonstrated the position-dependent cleavage upon PS substitutions. For clarity, the relative ratios of their initial cleavage velocities with respect to that of the un-substituted species are shown in Figure 4B-8B, and summarized in Table 3. For instance, PS substitutions at S6 position of PstI sequence (Figure 7B), and at S1 and S6 positions of SphI sequence (Figure 8B) led to an almost complete inhibition of the cleavage activity. In contrast, PS substitutions at S2, S3, S5, S6 and S7 of SpeI (Figure 4B), at S7 of XbaI (Figure 5B) and at S3 of SphI (Figure 8B) showed the enhanced cleavage.

**Table 3:** Relative initial cleavage rate of PS modification.

RV	DNA	PS1	PS2	PS3	PS4	PS5	PS6	PS7
EcoRV GAT/ATC	N	N	N	M	S	M	N	N
SpeI A/CTAGT	N	S	E	E	M	E	E	E
XbaI T/CTAGA	N	M	N	N	M	N	N	E
XhoI C/TCGAG	N	M	M	M	N	M	M	M
PstI CTGCA/G	N	N	M	N	S	S	I	M
SphI GCATG/C	N	I	N	E	N	M	I	S

EcoRV-S4, PstI-S6 and SphI-S6 have the PS substitutions just at the cleavage sites. These results could be interpreted by the fact that the replacement of oxygen atom by sulfur atom disturbs the direct interactions of the original oxygen atom with certain key amino acid residues in the catalytic centers and represses the cleavage consequently. Such observation has been reported for EcoRV, that substitution on only one strand by PS at the cleavage site has decreased the hydrolysis rate significantly [31]. On the other hand, SphI-S1 and SpeI-S1 are not at the cleavage sites, and these reduced cleavage efficiencies might be primarily attributed to the interference of the endonuclease-substrate binding.

Substitutions at the flanking 3′- and 5′-phosphate showed a wide variety in cleavage efficiency: some substitutions increased cleavage efficiency, such as XbaI-S7 and SpeI-S7, while others reduced cleavage efficiency greatly, such as XhoI-S1, SpeI-S1, SphI-S1 and SphI-S7. It was also observed that the pair of isocaudomers, SpeI and XbaI, did not show a similar cleavage pattern in spite of they can create the identical 5′-overhang.

The recognition of the substrate sequence by a restriction endonuclease depends on the direct contacts between each nucleotide of the recognition sequence and the relevant amino acid residues of endonucleases. The sulfur replacement affects the electron density on the phosphate group, leading to the changes in the cleavage activity. It should be emphasized that the phosphorothioate is a chiral group, showing different orientations of the P-S and P-O bonds in space, forming different hydrogen bond networks and resulting in different cleavage effects. Previous studies have shown that these distinctive *R*_p_-form and *S*_p_-form indeed influence the EcoRI cleavage differently. In general, two different orientations of these chiral isomers present a fast and a slow catalytic process, respectively. In this study, the purchased synthetic phosphorothioate is a mixture of *R*_p_-form and *S*_p_-form diastereomers, and our results could be the fastest one among the *R*_p_-form cleavage and *S*_p_-form cleavage. We have observed that some substitutions increased cleavage efficiencies, such as XbaI-S7, SpeI-S2, SpeI-S3, SpeI-S5, SpeI-S6 and SpeI-S7, being consistent with previous reports [4]. The exact mechanism for such enhancement has not been given. We speculate that some of the modifications may render the interaction of enzyme-substrate in a better pathway and might vary from enzyme to enzyme. More thorough analyses are needed.

### Comparison with Other Investigations

It has been proposed that the specific recognitions of cognate sequences by REs are attributed to both ‘direct readout’ and ‘indirect readout’. In the direct readout format, nucleobases in cognate sequences are recognized by the conserved amino acid residues, and in the case of indirect readout, the backbone of phosphate and sugar is held by structural element of enzymes. Many co-crystal structures of protein-DNA have shown the distorted conformation aberrant from canonical B-form DNA, such as EcoRI and EcoRV. The backbone distortion is believed to facilitate the optimal matching between these two macromolecules. Thus, indirect readout of the protein-backbone interactions also plays key role in specific recognition and cleavage. Consequently, phosphate and sugar modified analogues are significant tools for these studies.

EcoRV is one of the best characterized endonucleases. When forming a complex with its substrate, the dsDNA is positioned in a cleft between two monomers. The dsDNA makes contacts primarily with two peptide loops from each monomer as well as with other segments of EcoRV. The recognition loop, known as R loop, lies in the major groove of the DNA and makes several hydrogen bonds with bases of the recognition sequence. The Q loop, having two glutamines between residues 67 and 72, interacts extensively with the sugar–phosphate backbone in the minor groove, placing the phosphodiester bond in the active site of EcoRV. The crystal structures of the free EcoRV show that both the R loop and the Q loop are largely disordered [32]. In the process of cleavage, first, EcoRV binds to the dsDNA at a random sequence and slides along it until the two outer base pairs, GAXXTC, are recognized. Non-specific DNA binding is the prerequisite for this one-dimensional diffusion of enzyme along DNA. Then, the R-loop becomes more ordered, leading to the formation of strong hydrogen-bonding interactions and a partially bound EcoRV-DNA complex. Finally, the center nucleotides are probed, and the dsDNA bends about 50 degree at the -T3pA4- phosphate. This bending leads to an unstacking of the bases, widening of the minor groove with a concomitant compression of the major groove, which brings the scissile phosphate oxygen deeper into the active site to accomplish the cleavage [33], [34], [35]. Based on this model, the two outer base pairs are responsible for the binding and the central base pairs are for the cleavage. Thus, it is understandable that the central nucleotides are more sensitive to the analogue substitutions, resulting in the U-shaped position-dependent cleavage efficiency (Figure 3B and 3D).

Thorogood *et al.* have used phosphorothioate isomers (*R*_p_ and *S*_p_) to detail the interaction networks of EcoRV-DNA. The d(GACGATA-*S*_p_-TCGTC) has been found not to be a substrate, while the d(GACGATA-*R*_p_-TCGTC) has been well hydrolyzed [5]. For the *R*_p_ phosphorothioate an S atom replaces the pro-R oxygen, which is able to deprotonate the attacking water molecule. In the case of the *S*_p_ phosphorothioate, an uncharged double-bonded oxygen is placed in the *R*_p_ position. Interestingly, most of the phosphorothioate substituted substrates bound more tightly to the endonuclease as shown by the decreased K_m_ [5]. Jeltsch *et al.* have also investigated the cleavage rates of phosphorothioate substitutions of d(GACGATATCGTC) sequence with EcoRV in Mg^2+^ or Mn^2+^ solution [36] . The cleavage rate of the d(GACGATA-*S*_p_-TCGTC) was reduced by a factor of >10^6^ whereas the cleavage of d(GACGATA-*R*_p_-TCGTC) was retarded by only 2 folds in the presence of Mg^2+^. In addition, slightly enhanced cleavage rates were observed for d(GA-*R*_p_-CGATATCGTC) and d(GAC-*S*_p_-GATATCGTC) [36] . Our study showed that *-*T3pA4- was the most inhibited PS modification position for cleavage. This is mainly attributed to the weakening of Mg^2+^ binding non-bridging oxygen of phosphate group in cleavage site and inhibiting the right assembling of catalytic center. -A2pT3- and -A4pT5- substitutions were also poor substrates. These two phosphates were suggested to interact with two key amino acids residues: Thr93 and Ser112 [37], and breaking down of their interactions may further disturb the elaborated hydrogen bond networks and ionic interactions involved in catalysis. Other PS substitution positions of our study showed little effects on cleavage efficiency. In comparison with previous studies, the relatively moderate reduction in our study might be attributed to the single strand rather than double strand modification and the mixed isomers of phosphorothioate. Our data are consistent with the previous studies that single strand substitution of the cleavage site by a phosphorothioate residue decreases the EcoRV-catalyzed hydrolysis rate significantly but not completely [31]. On the whole, our results demonstrate a good consistence with previous studies, since all of these results have showed that PS substitutions bring the most reduction in vicinity to the EcoRV cleavage site while are tolerated at adjacent sequences.

In this study, we also carried out 2′-OMeN substitution of EcoRV cleavage for the first time. In comparison with PS substitution, 2′-OMeN behave similar position-dependent manner which was also U-shaped but to a stronger extent. This implies that the perturbation of the interaction with non-bridging oxygen and the consequent effects might be common explanation for both of the two substitutions. Based on the crystal structure of EcoRV-DNA complex (1RVB), we also analyzed each C2′ position in cognate sequence. Except A2, most of the distances to nearby functional elements of amino acid residues were more than 5 Å which reduced possibility of affecting directly by 2′-OMe group. The C2′ of A2 is in close vicinity to Asn70 and Asn120 with the nearest distances of 4.07 Å and 3.56 Å respectively. However, the side chains of Asn are not proposed to have a significant interaction with O-methyl group in such a distance. Therefore, regarding 2′-OMeN substitution, the position-dependent inhibitory effect may probably arise from backbone change by C3′-endo pucker conformation. We also should noticed that the consensus characteristic of PS and 2′-OMeN substitutions in EcoRV is an individual rather than a universal phenomenon, since we can not find the second coincidence in the other five REs of our study, which suggested that, except of phosphate backbone changes, more complex alterations induced by 2′-OMeN substitution were involved for these REs. The only reported C2′ modification in EcoRV cleavage was 2′-fluoro, and substitutions of C2′-fluoro nucleotide at T3 and T5 positions have decreased velocities [12], which is in a good agreement with our 2′-OMeN results.

Interpretation of the cleavage efficiency variations of other five endonucleases needs a more analytical work since no structural details are available at this moment. Further analyses showed that 2′-OMeN substitutions at non-cleavage positions can still affect the cleavage efficiency significantly, such as SpeI-T6, XbaI-T3, XbaI-A4, XhoI-G4, XhoI-A5, XhoI-G6, PstI-C1, PstI-T2, PstI-G3, PstI-C4, SphI-A3 and SphI-T4. All these data indicate that it is not necessary to make a substitution at the cleavage site for cleavage protection, since 2′-OMeN can offer a more flexible choice of positions for substitution. On the other hand, both 2′-OMeN and PS substitutions at several positions could enhance the enzymatic cleavage, which has a great potential in bioengineering applications.

### Biological Significance

The protective effect of nucleotide substitutions observed in this study could have potential applications in many aspects of life science and medicine. Mutation in the mitochondrial DNA (mtDNA) can cause human diseases. Usually, such mutations are heteroplasmic (*i. e.* mutated mtDNA and wild-type mtDNA coexist), and a small percentage of wild-type sequences can have a strong protective effect against the metabolic defect [38]. In the experiment conducted by Srivastava and Moraes, PstI gene has been constructed and efficiently expressed in human cells. Its product has been transformed into mitochondria efficiently, where the mitochondrial PstI targets the mtDNA haplotype harboring PstI sites. In contrast, in a heteroplasmic environment, a mtDNA haplotype lacking PstI sites has been preferentially maintained, causing a significant shift in heteroplasma [16]. Their experiments provide a proof of the principle that restriction endonucleases are feasible tools for genetic therapy of a sub-group of mitochondrial disorders, although this approach is very preliminary. We expect that the nucleotide analogue substitutions could accomplish the similar functions for treatment of such DNA mutation in the future.

High-throughput genomics and the emerging field of synthetic biology demand more convenient, economical, and efficient technologies to clone genes, to assemble gene libraries and to identify synthetic pathways. Although current cloning technologies based on site-specific recombination are simple, efficient and flexible, they have drawbacks of leaving recombination site sequences and adding an extra 8 to 13 amino acids to the expressed protein consequently. Engler *et al.* have developed a protocol to assemble, in one step and one tube, at least nine separate DNA fragments together into an acceptor vector. This protocol is based on the use of type II restriction endonucleases and is performed by simply subjecting a mix of 10 undigested input plasmids (nine insert plasmids and the acceptor vector) to a restriction-ligation and transforming the resulting mix in competent cells [39]. This approach can also cut outside of their recognition sequence, and the ligated products lack the original restriction site [40]. It is our hope that using appropriate nucleotide analogue substitutions at different recognition sites might create a variety of cleavage patterns and benefit the development of cloning approaches in synthetic biology.

## Conclusions

In this study, we examined the effects of nucleotide analogues (2′-O-methyl and phosphorothioate) substitutions on the cleavage efficiency of six type II restriction endonucleases. Experimental results clearly showed that nucleotide substitutions can change the enzymatic cleavage in a position-dependent manner. In most cases, substitutions decreased or even inhibited the cleavage completely, demonstrating a protective behavior. On the other hand, substitutions at other positions could enhance the phosphoester bond hydrolysis. Rational interpretations for these altered endonuclease-catalyzed cleavages are under further study. It is our hope that nucleotide analogue substitutions could be used wisely for protection of unwanted cleavage of the native host genome as well as effective digestion of the invading foreign nucleic acids.

## Supporting Information

Figure S1 **Effects of enzyme concentrations and position-dependent substitution on SpeI cleavage.** (A) Time course plot of fluorescence intensity affected by enzyme concentrations. (B) Initial velocities affected by enzyme concentrations. (C) Time course plot of fluorescence intensity affected by 2′-OMeN substitution positions. (D) Time course plot of fluorescence intensity affected by PS substitution positions.
(TIF)

Figure S2 **Effects of enzyme concentrations and position-dependent substitution on XbaI cleavage.** (A) Time course plot of fluorescence intensity affected by enzyme concentrations. (B) Initial velocities affected by enzyme concentrations. (C) Time course plot of fluorescence intensity affected by 2′-OMeN substitution positions. (D) Time course plot of fluorescence intensity affected by PS substitution positions.
(TIF)

Figure S3 **Effects of enzyme concentrations and position-dependent substitution on XhoI cleavage.** (A) Time course plot of fluorescence intensity affected by enzyme concentrations. (B) Initial velocities affected by enzyme concentrations. (C) Time course plot of fluorescence intensity affected by 2′-OMeN substitution positions. (D) Time course plot of fluorescence intensity affected by PS substitution positions.
(TIF)

Figure S4 **Effects of enzyme concentrations and position-dependent substitution on PstI cleavage.** (A) Time course plot of fluorescence intensity affected by enzyme concentrations. (B) Initial velocities affected by enzyme concentrations. (C) Time course plot of fluorescence intensity affected by 2′-OMeN substitution positions. (D) Time course plot of fluorescence intensity affected by PS substitution positions.
(TIF)

Figure S5 **Effects of enzyme concentrations and position-dependent substitution on SphI cleavage.** (A) Time course plot of fluorescence intensity affected by enzyme concentrations. (B) Initial velocities affected by enzyme concentrations. (C) Time course plot of fluorescence intensity affected by 2′-OMeN substitution positions. (D) Time course plot of fluorescence intensity affected by PS substitution positions.
(TIF)

Table S1 **Reported nucleotide analog studies on five REs cleavage.** *D: double-stranded substitution; S: single-stranded substitution; N: no cleavage; Y: cleavage; P: partial cleavage.
(DOC)

## References

[1] Roberts RJ (1982) Restriction and modification enzymes and their recognition sequences. Nucleic Acids Res 10: r117–144. 6280143 10.1093/nar/10.5.1770 PMC320569

[2] Pingoud A, Jeltsch A (2001) Structure and function of type II restriction endonucleases. Nucleic Acids Res 29: 3705–3727. 11557805 10.1093/nar/29.18.3705 PMC55916

[3] Roberts RJ (1990) Restriction enzymes and their isoschizomers. Nucleic Acids Res 18 Suppl: 2331–2365 10.1093/nar/18.suppl.2331 PMC331877 2159140

[4] Mazurek M, Sowers LC (1996) The paradoxical influence of thymine analogues on restriction endonuclease cleavage of oligodeoxynucleotides. Biochemistry 35: 11522–11528. 8784209 10.1021/bi953012j

[5] Thorogood H, Grasby JA, Connolly BA (1996) Influence of the phosphate backbone on the recognition and hydrolysis of DNA by the EcoRV restriction endonuclease. A study using oligodeoxynucleotide phosphorothioates. J Biol Chem 271: 8855–8862. 8621526 10.1074/jbc.271.15.8855

[6] Crouzier L, Dubois C, Wengel J, Veedu RN (2012) Cleavage and protection of locked nucleic acid-modified DNA by restriction endonucleases. Bioorg Med Chem Lett 22: 4836–4838. 22727669 10.1016/j.bmcl.2012.05.113

[7] Macickova-Cahova H, Hocek M (2009) Cleavage of adenine-modified functionalized DNA by type II restriction endonucleases. Nucleic Acids Res 37: 7612–7622. 19820117 10.1093/nar/gkp845 PMC2794189

[8] Newman PC, Nwosu VU, Williams DM, Cosstick R, Seela F, et al (1990) Incorporation of a complete set of deoxyadenosine and thymidine analogues suitable for the study of protein nucleic acid interactions into oligodeoxynucleotides. Application to the EcoRV restriction endonuclease and modification methylase. Biochemistry 29: 9891–9901. 2271627 10.1021/bi00494a020

[9] Waters TR, Connolly BA (1994) Interaction of the restriction endonuclease EcoRV with the deoxyguanosine and deoxycytidine bases in its recognition sequence. Biochemistry 33: 1812–1819. 8110783 10.1021/bi00173a026

[10] Nielsen PE, Egholm M, Berg RH, Buchardt O (1993) Sequence specific inhibition of DNA restriction enzyme cleavage by PNA. Nucleic Acids Res 21: 197–200. 8382793 10.1093/nar/21.2.197 PMC309092

[11] Sayers JR, Olsen DB, Eckstein F (1989) Inhibition of restriction endonuclease hydrolysis by phosphorothioate-containing DNA. Nucleic Acids Res 17: 9495. 2555795 10.1093/nar/17.22.9495 PMC335172

[12] Williams DM, Benseler F, Eckstein F (1991) Properties of 2′-fluorothymidine-containing oligonucleotides: interaction with restriction endonuclease EcoRV. Biochemistry 30: 4001–4009. 2018768 10.1021/bi00230a027

[13] Li J, Deng T, Chu X, Yang R, Jiang J, et al (2010) Rolling circle amplification combined with gold nanoparticle aggregates for highly sensitive identification of single-nucleotide polymorphisms. Anal Chem 82: 2811–2816. 20192245 10.1021/ac100336n

[14] Liu X-P, Liu J-H (2010) The terminal 5′ phosphate and proximate phosphorothioate promote ligation-independent cloning. Protein Science 19: 967–973. 20217896 10.1002/pro.374 PMC2868239

[15] Howland SW, Poh C-M, Renia L (2011) Directional, seamless, and restriction enzyme-free construction of random-primed complementary DNA libraries using phosphorothioate-modified primers. Analytical Biochemistry 416: 141–143. 21530478 10.1016/j.ab.2011.04.006

[16] Srivastava S, Moraes CT (2001) Manipulating mitochondrial DNA heteroplasmy by a mitochondrially targeted restriction endonuclease. Hum Mol Genet 10: 3093–3099. 11751691 10.1093/hmg/10.26.3093

[17] Kawasaki AM, Casper MD, Freier SM, Lesnik EA, Zounes MC, et al (1993) Uniformly modified 2′-deoxy-2′-fluoro phosphorothioate oligonucleotides as nuclease-resistant antisense compounds with high affinity and specificity for RNA targets. J Med Chem 36: 831–841. 8464037 10.1021/jm00059a007

[18] Freier SM, Altmann KH (1997) The ups and downs of nucleic acid duplex stability: structure-stability studies on chemically-modified DNA:RNA duplexes. Nucleic Acids Res 25: 4429–4443. 9358149 10.1093/nar/25.22.4429 PMC147101

[19] Pallan PS, Greene EM, Jicman PA, Pandey RK, Manoharan M, et al (2011) Unexpected origins of the enhanced pairing affinity of 2′-fluoro-modified RNA. Nucleic Acids Res 39: 3482–3495. 21183463 10.1093/nar/gkq1270 PMC3082899

[20] Piao X, Sun L, Zhang T, Gan Y, Guan Y (2008) Effects of mismatches and insertions on discrimination accuracy of nucleic acid probes. Acta Biochim Pol 55: 713–720. 19039337

[21] Campbell JM, Bacon TA, Wickstrom E (1990) Oligodeoxynucleoside phosphorothioate stability in subcellular extracts, culture media, sera and cerebrospinal fluid. J Biochem Biophys Methods 20: 259–267. 2188993 10.1016/0165-022x(90)90084-p

[22] Phillips MI, Zhang YC (2000) Basic principles of using antisense oligonucleotides in vivo. Methods Enzymol 313: 46–56. 10595348 10.1016/s0076-6879(00)13004-6

[23] Majlessi M, Nelson NC, Becker MM (1998) Advantages of 2′-O-methyl oligoribonucleotide probes for detecting RNA targets. Nucleic Acids Res 26: 2224–2229. 9547284 10.1093/nar/26.9.2224 PMC147516

[24] Dominick PK, Jarstfer MB (2004) A conformationally constrained nucleotide analogue controls the folding topology of a DNA g-quadruplex. J Am Chem Soc 126: 5050–5051. 15099071 10.1021/ja039192z

[25] Frey PA, Sammons RD (1985) Bond order and charge localization in nucleoside phosphorothioates. Science 228: 541–545. 2984773 10.1126/science.2984773

[26] Li X, Song C, Zhao M, Li Y (2008) Continuous monitoring of restriction endonuclease cleavage activity by universal molecular beacon light quenching coupled with real-time polymerase chain reaction. Anal Biochem 381: 1–7. 18611388 10.1016/j.ab.2008.06.027

[27] Urata H, Tamaki C, Matsuno M, Wada S, Akagi M (2009) FRET-based kinetic analysis of highly reactive heterochiral DNA toward EcoRI endonuclease. Biochem Biophys Res Commun 390: 192–195. 19732747 10.1016/j.bbrc.2009.08.164

[28] Huang Y, Zhao S, Shi M, Chen J, Chen ZF, et al (2011) Intermolecular and intramolecular quencher based quantum dot nanoprobes for multiplexed detection of endonuclease activity and inhibition. Anal Chem 83: 8913–8918. 22017679 10.1021/ac2013114

[29] Eisenschmidt K, Lanio T, Jeltsch A, Pingoud A (2002) A fluorimetric assay for on-line detection of DNA cleavage by restriction endonucleases. J Biotechnol 96: 185–191. 12039534 10.1016/s0168-1656(02)00029-9

[30] Yan Y, Yan J, Piao X, Zhang T, Guan Y (2012) Effect of LNA- and OMeN-modified oligonucleotide probes on the stability and discrimination of mismatched base pairs of duplexes. J Biosci 37: 233–241. 22581329 10.1007/s12038-012-9196-4

[31] Olsen DB, Kotzorek G, Eckstein F (1990) Investigation of the inhibitory role of phosphorothioate internucleotidic linkages on the catalytic activity of the restriction endonuclease EcoRV. Biochemistry 29: 9546–9551. 2271600 10.1021/bi00493a008

[32] Perona JJ, Martin AM (1997) Conformational transitions and structural deformability of EcoRV endonuclease revealed by crystallographic analysis. J Mol Biol 273: 207–225. 9367757 10.1006/jmbi.1997.1315

[33] Fliess A, Wolfes H, Seela F, Pingoud A (1988) Analysis of the recognition mechanism involved in the EcoRV catalyzed cleavage of DNA using modified oligodeoxynucleotides. Nucleic Acids Res 16: 11781–11793. 3062581 10.1093/nar/16.24.11781 PMC339110

[34] Fliess A, Wolfes H, Rosenthal A, Schwellnus K, Blocker H, et al (1986) Role of thymidine residues in DNA recognition by the EcoRI and EcoRV restriction endonucleases. Nucleic Acids Res 14: 3463–3474. 3010238 10.1093/nar/14.8.3463 PMC339785

[35] Hancox EL, Connolly BA, Walker RT (1993) Synthesis and properties of oligodeoxynucleotides containing the analogue 2′-deoxy-4′-thiothymidine. Nucleic Acids Res 21: 3485–3491. 8346027 10.1093/nar/21.15.3485 PMC331449

[36] Jeltsch A, Maschke H, Selent U, Wenz C, Kohler E, et al (1995) DNA binding specificity of the EcoRV restriction endonuclease is increased by Mg2+ binding to a metal ion binding site distinct from the catalytic center of the enzyme. Biochemistry 34: 6239–6246. 7742329 10.1021/bi00018a028

[37] Wenz C, Jeltsch A, Pingoud A (1996) Probing the indirect readout of the restriction enzyme EcoRV. Mutational analysis of contacts to the DNA backbone. J Biol Chem 271: 5565–5573. 8621416 10.1074/jbc.271.10.5565

[38] Moraes CT, Ricci E, Petruzzella V, Shanske S, DiMauro S, et al (1992) Molecular analysis of the muscle pathology associated with mitochondrial DNA deletions. Nat Genet 1: 359–367. 1284549 10.1038/ng0892-359

[39] Engler C, Gruetzner R, Kandzia R, Marillonnet S (2009) Golden gate shuffling: a one-pot DNA shuffling method based on type IIs restriction enzymes. PLoS One 4: e5553. 19436741 10.1371/journal.pone.0005553 PMC2677662

[40] Engler C, Kandzia R, Marillonnet S (2008) A one pot, one step, precision cloning method with high throughput capability. PLoS One 3: e3647. 18985154 10.1371/journal.pone.0003647 PMC2574415

