# Supplementary figures and images for: Enzymatic Cleavage of Type II Restriction Endonucleases on the 2′-O-Methyl Nucleotide and Phosphorothioate Substituted DNA
Source: PLoS One. 2013 Nov 15;8(11):e79415. doi: 10.1371/journal.pone.0079415 (PMC3829850; doi:10.1371/journal.pone.0079415)

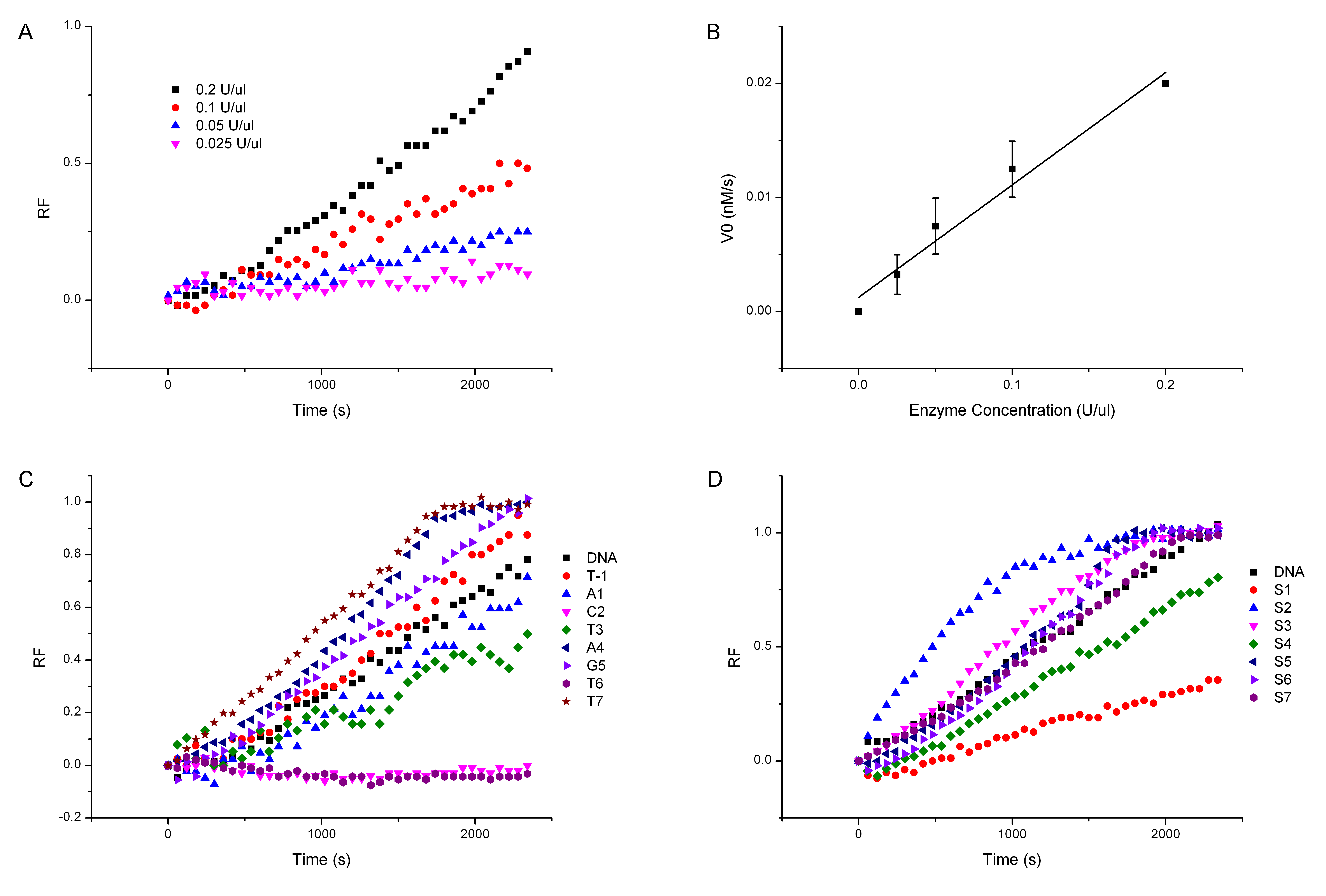

Supplement: Figure S1 — Effects of enzyme concentrations and position-dependent substitution on SpeI cleavage. (A) Time course plot of fluorescence intensity affected by enzyme concentrations. (B) Initial velocities affected by enzyme concentrations. (C) Time course plot of fluorescence intensity affected by 2′-OMeN substitution positions. (D) Time course plot of fluorescence intensity affected by PS substitution positions. (TIF) [file pone.0079415.s001.tif]

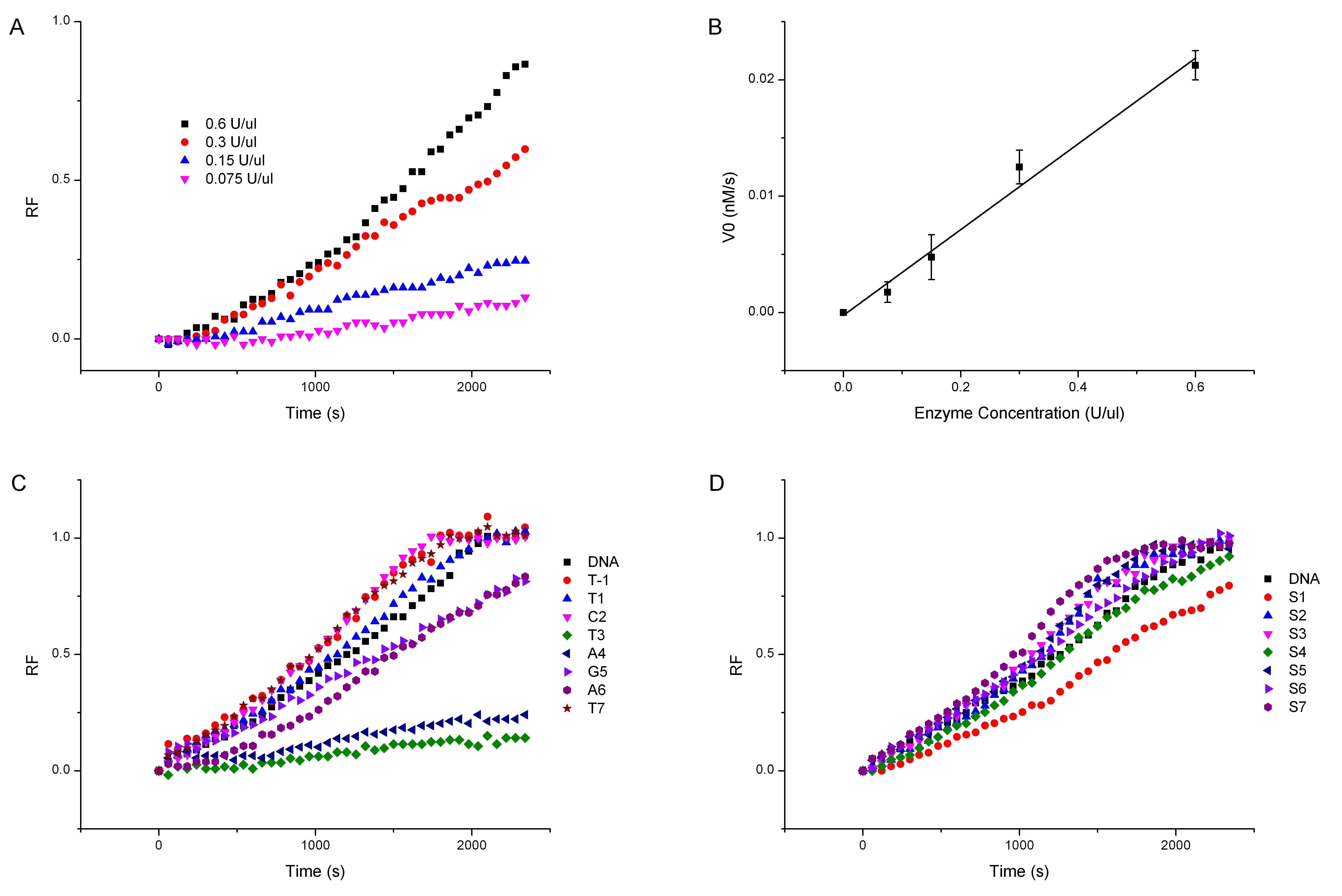

Supplement: Figure S2 — Effects of enzyme concentrations and position-dependent substitution on XbaI cleavage. (A) Time course plot of fluorescence intensity affected by enzyme concentrations. (B) Initial velocities affected by enzyme concentrations. (C) Time course plot of fluorescence intensity affected by 2′-OMeN substitution positions. (D) Time course plot of fluorescence intensity affected by PS substitution positions. (TIF) [file pone.0079415.s002.tif]

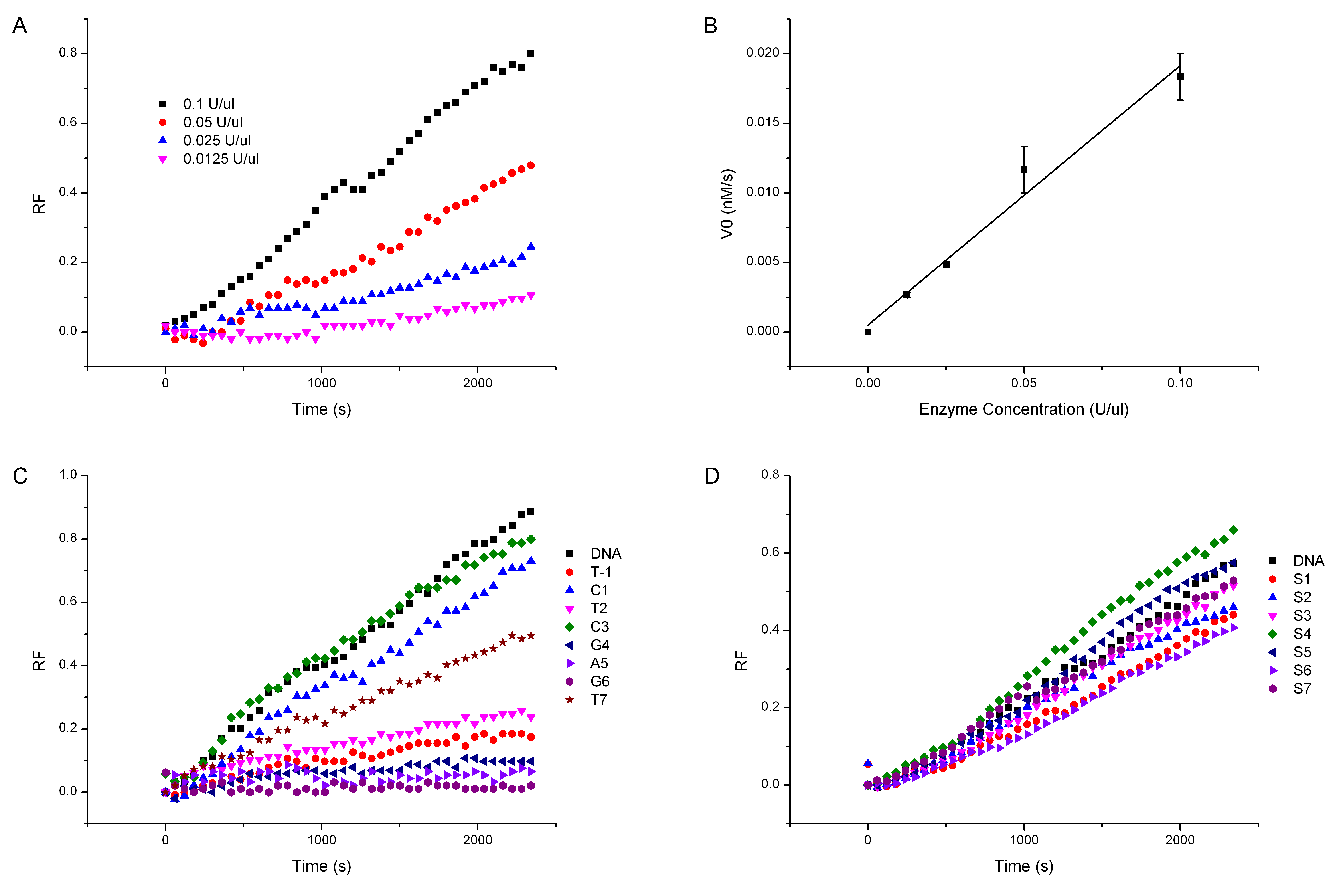

Supplement: Figure S3 — Effects of enzyme concentrations and position-dependent substitution on XhoI cleavage. (A) Time course plot of fluorescence intensity affected by enzyme concentrations. (B) Initial velocities affected by enzyme concentrations. (C) Time course plot of fluorescence intensity affected by 2′-OMeN substitution positions. (D) Time course plot of fluorescence intensity affected by PS substitution positions. (TIF) [file pone.0079415.s003.tif]

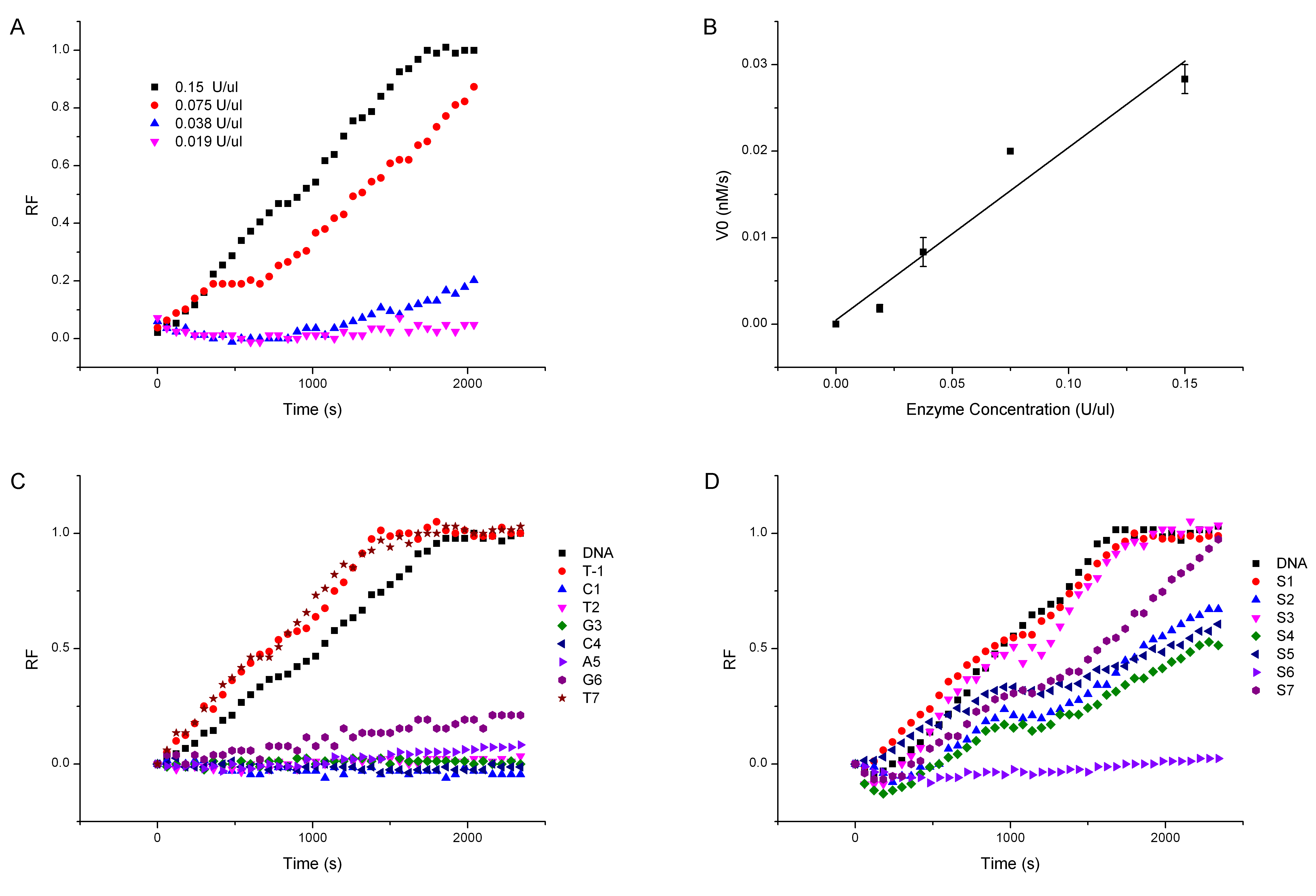

Supplement: Figure S4 — Effects of enzyme concentrations and position-dependent substitution on PstI cleavage. (A) Time course plot of fluorescence intensity affected by enzyme concentrations. (B) Initial velocities affected by enzyme concentrations. (C) Time course plot of fluorescence intensity affected by 2′-OMeN substitution positions. (D) Time course plot of fluorescence intensity affected by PS substitution positions. (TIF) [file pone.0079415.s004.tif]

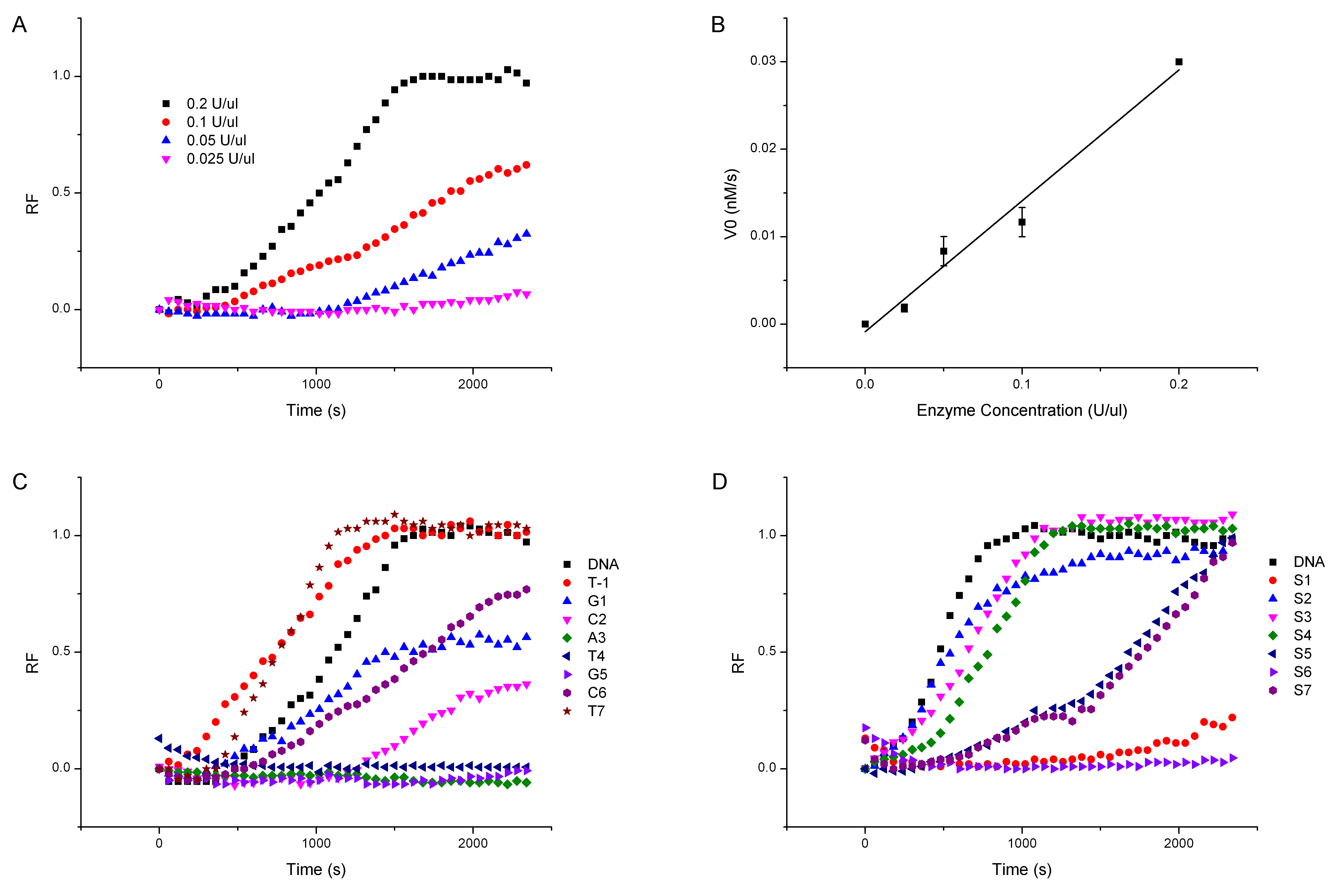

Supplement: Figure S5 — Effects of enzyme concentrations and position-dependent substitution on SphI cleavage. (A) Time course plot of fluorescence intensity affected by enzyme concentrations. (B) Initial velocities affected by enzyme concentrations. (C) Time course plot of fluorescence intensity affected by 2′-OMeN substitution positions. (D) Time course plot of fluorescence intensity affected by PS substitution positions. (TIF) [file pone.0079415.s005.tif]
