# Supplementary material for: Enzymatic Cleavage of Type II Restriction Endonucleases on the 2′-O-Methyl Nucleotide and Phosphorothioate Substituted DNA
Source: PLoS One. 2013 Nov 15;8(11):e79415. doi: 10.1371/journal.pone.0079415 (PMC3829850; doi:10.1371/journal.pone.0079415)
Supplement: Table S1 — Reported nucleotide analog studies on five REs cleavage. *D: double-stranded substitution; S: single-stranded substitution; N: no cleavage; Y: cleavage; P: partial cleavage. (DOC) [file pone.0079415.s006.doc]

**Supplementary materials:**

**Table S1. Reported nucleotide analog studies on five REs cleavage.**

| Enzyme | Nucleotide analog | Substituted position | Substitution strand* | Effects of modification* | Reference |
| --- | --- | --- | --- | --- | --- |
|  | 5Me | C2 | D | N |  |
| SpeI | 5Me | C2 | S | N |  |
|  | Me | A1 | S | N |  |
|  | 5Me | C2 | S | N |  |
|  | 5Me | C2 | D | N |  |
|  | 5GHMe | C2 | D | N |  |
| XbaI | 5PGHP | T1 and T3 | D | N |  |
|  | 5HMe | T1 and T3 | D | P |  |
|  | 5H | T1 and T3 | D | P |  |
|  | 7deaza | G5 | D | Y |  |
|  | 5Me | C1 and C3 | S | N |  |
|  | U | T2 | S | I |  |
| XhoI | 5HMeU | T2 | S | P |  |
|  | 6Me | G4 | S | P |  |
|  | 6Me | G6 | S | P |  |
|  | 5Me | C1 and C4 | S | N |  |
|  | 5Me | C1 and C4 | S | N |  |
|  | 5Hg | C1 and C4 | S | N |  |
|  | 5E | C1 and C4 | S | N |  |
|  | 5Ph | C1 and C4 | S | N |  |
|  | UE | T2 | S | N |  |
|  | Uph | T2 | S | N |  |
|  | 7deaza | G3 and G6 | D | N |  |
|  | 7deaza | G3,G6 and G7 | D | N |  |
| PstI | 7deaza | A5 | D | Y |  |
|  | 7H | A5 | S | Y |  |
|  | 7E | A5 | S | N |  |
|  | 7Ph | A5 | S | N |  |
|  | 7N0_2_ | A5 | S | N |  |
|  | 8Br | A5 | S | Y |  |
|  | 8Me | A5 | S | Y |  |
|  | LNA | A5 | S | P |  |
|  | LNA | A5 | D | N |  |
|  | PS | A5pG6 | S | P |  |
|  | 5E | C2 and C6 | S | N |  |
|  | 5Ph | C2 and C6 | S | N |  |
|  | UE | T4 | S | P |  |
|  | Uph | T4 | S | N |  |
|  | 7deaza | G1 and G5 | D | N |  |
| SphI | 7deaza | A3 | D | P |  |
|  | 7H | A3 | S | Y |  |
|  | 7E | A3 | S | P |  |
|  | 7Ph | A3 | S | N |  |
|  | 7N0_2_ | A3 | S | N |  |
|  | 8Br | A3 | S | P |  |
|  | 8Me | A3 | S | P |  |

*D: double-stranded substitution; S: single-stranded substitution; N: no cleavage; Y: cleavage; P: partial cleavage.

References

1. Wong KK, McClelland M (1991) PCR with 5-methyl-dCTP replacing dCTP. Nucleic Acids Res 19: 1081-1085.

2. Nelson PS, Papas TS, Schweinfest CW (1993) Restriction endonuclease cleavage of 5-methyl-deoxycytosine hemimethylated DNA at high enzyme-to-substrate ratios. Nucleic Acids Res 21: 681-686.

3. Hofer B (1988) The sensitivity of DNA cleavage by SpeI and ApaLI to methylation by M.EcoK. Nucleic Acids Res 16: 5206.

4. Huang LH, Farnet CM, Ehrlich KC, Ehrlich M (1982) Digestion of highly modified bacteriophage DNA by restriction endonucleases. Nucleic Acids Res 10: 1579-1591.

5. Grime SK, Martin RL, Holaway BL (1991) Inhibition of restriction enzyme cleavage of DNA modified with 7-deaza-dGTP. Nucleic Acids Res 19: 2791.

6. Mazurek M, Sowers LC (1996) The paradoxical influence of thymine analogues on restriction endonuclease cleavage of oligodeoxynucleotides. Biochemistry 35: 11522-11528.

7. Voigt JM, Topal MD (1990) O6-methylguanine in place of guanine causes asymmetric single-strand cleavage of DNA by some restriction enzymes. Biochemistry 29: 1632-1637.

8. Gruenbaum Y, Cedar H, Razin A (1981) Restriction enzyme digestion of hemimethylated DNA. Nucleic Acids Res 9: 2509-2515.

9. Banfalvi G, Sarkar N (1995) Effect of mercury substitution of DNA on its susceptibility to cleavage by restriction endonucleases. DNA Cell Biol 14: 445-450.

10. Macickova-Cahova H, Pohl R, Hocek M (2011) Cleavage of functionalized DNA containing 5-modified pyrimidines by type II restriction endonucleases. Chembiochem 12: 431-438.

11. Seela F, Roling A (1992) 7-Deazapurine containing DNA: efficiency of c7GdTP, c7AdTP and c7IdTP incorporation during PCR-amplification and protection from endodeoxyribonuclease hydrolysis. Nucleic Acids Res 20: 55-61.

12. Macickova-Cahova H, Hocek M (2009) Cleavage of adenine-modified functionalized DNA by type II restriction endonucleases. Nucleic Acids Res 37: 7612-7622.

13. Crouzier L, Dubois C, Wengel J, Veedu RN (2012) Cleavage and protection of locked nucleic acid-modified DNA by restriction endonucleases. Bioorg Med Chem Lett 22: 4836-4838.

14. Taylor JW, Schmidt W, Cosstick R, Okruszek A, Eckstein F (1985) The use of phosphorothioate-modified DNA in restriction enzyme reactions to prepare nicked DNA. Nucleic Acids Res 13: 8749-8764.
